# Association between cognitive function and skeletal muscle in patients undergoing maintenance hemodialysis

**DOI:** 10.3389/fendo.2024.1324867

**Published:** 2024-03-15

**Authors:** Lulu Wang, Xueqin Bian, Lilin Liu, Qingyun He, Jie Xu, Xue Chen, Hong Ye, Junwei Yang, Lei Jiang

**Affiliations:** Center for Kidney Disease, The Second Affiliated Hospital, Nanjing Medical University, Nanjing, Jiangsu, China

**Keywords:** cognitive function, skeletal muscle, BDNF, hemodialysis, MoCA

## Abstract

**Background:**

Patients on hemodialysis have a higher burden of cognitive impairment than individuals of the same age in the general population. Studies have found a link between cognition and skeletal muscle function. However, few studies have investigated these associations and the underlying mechanisms in patients on hemodialysis.

**Methods:**

A total of 166 patients on hemodialysis were enrolled in this longitudinal study. Cognitive function was assessed by Montreal Cognitive Assessment (MoCA) scores. Skeletal muscle indicators were evaluated using Inbody S10. Plasma brain-derived neurotrophic factor (BDNF) concentrations were measured by enzyme-linked immunosorbent assay. The primary outcome was a change in the MoCA scores. A mediation analysis was performed to examine the indirect effect of skeletal muscle on cognitive decline through BDNF.

**Results:**

Among the 166 patients, the average age was 49.9 ± 11.2 years. Of these patients with a median follow-up of 1,136 days, 133 participated in the study. We defined MoCA scores decreased by ≥2 points at 3 years from the baseline measurement as cognitive decline (CD). Compared to the cognitively unchanged group, patients with CD had significantly lower fat-free mass, soft lean mass, skeletal muscle mass, and skeletal muscle index (all P<0.05). After adjusting for potential confounders, skeletal muscle indicators were protective predictors of CD. A significant increase in plasma BDNF levels was observed in the CD group. Mediation analysis suggested that BDNF played a mediating role of 20-35% between cognitive impairment and skeletal muscle.

**Conclusion:**

Skeletal muscle is a protective predictor of CD in patients undergoing dialysis. BDNF mediates the relationship between cognitive impairment and skeletal muscle function.

## Introduction

Patients with chronic kidney disease (CKD) have a significantly higher risk for cognitive impairment than the general population. Renal failure is an independent risk factor for developing cognitive impairment and dementia. The prevalence of cognitive impairment ranges from approximately 10% to 40% in patients with different stages of CKD and is as high as 80% in patients undergoing hemodialysis (1, 2). Karakizlis et al. assessed the cognitive function of 408 patients who underwent regular hemodialysis and found that only 25% had normal cognitive function, whereas 50.5% had mild to moderate cognitive impairment and 24.5% had severe cognitive impairment (3).

The pathophysiological mechanisms by which cognitive impairment occurs in patients undergoing hemodialysis is complex and includes various factors related to vascular diseases, conventional cardiovascular factors, and dialysis (4). Previous studies have confirmed that crosstalk occurs between the skeletal muscles and brain (5). A study involving 281 elderly male patients confirmed a significant correlation between the radial and tibial muscle densities and cognitive function (6). The mechanisms underlying this correlation are important to elucidate.

Brain-derived neurotrophic factor (BDNF) is a member of the neurotrophin family that promotes neuronal development, cell survival, neurotransmission, and the maintenance of energy homeostasis. BDNF is expressed primarily in the hippocampus and cortex of the brain as well as in skeletal muscles (7). BDNF levels in the brain can enter the peripheral blood through the blood-brain barrier, and these levels are positively correlated with changes in serum BDNF levels. Therefore, peripheral BDNF levels may reflect changes in BDNF levels in the brain (8). Altered BDNF levels are associated with increased appetite, obesity, type 2 diabetes, schizophrenia, depression, and neurodegenerative diseases, including Alzheimer’s disease (9). Both BDNF-mediated synaptogenesis and neurogenesis contribute to enhanced cognition (10). Running and other types of aerobic exercises increase serum BDNF concentrations and enhance cognitive performance in human subjects (11).

This study aimed to analyze the correlation between changes in cognitive level and skeletal muscle mass in patients undergoing hemodialysis and explore the possible mechanism by which BDNF mediates skeletal muscle mass and cognitive disorders through a mediation analysis.

## Methods

### Study population

We conducted a prospective cohort study of patients received maintenance hemodialysis (HD) in the Second Affiliated Hospital of Nanjing Medical University. A total of 166 patients were enrolled on the non-dialysis day following the second dialysis treatment in a week. Inclusion criteria included participants who age ≥ 18 years and all participants had been on HD three times per week for 4h over 3 months. Exclusion criteria included: (1) Previous history of neurologic diseases (self-reported or medical records); (2) plans to transfer to other dialysis centers or receive kidney transplant within 1 year; (3) acute infection or any other condition that precludes the patients from immediate participation; (4) illiteracy, visual impairment or any other cause that leads to the inability to complete cognitive tests; (5) contraindications to magnetic resonance imaging; (6) malignancy with life expectancy <1 year. The study was reviewed and approved by the institutional research ethics committee of The Second Affiliated Hospital of Nanjing Medical University, and all participants provided written informed consent.

### Data and sample acquisition

Interview questionnaire was administered by trained research staff on demographic and medical information. Routine clinical laboratory tests were performed on the morning of the day of enrollment.

### Body composition assessment

A multi-frequency bioelectrical impedance analyzer, InBody S10 (Biospace, Seoul, Korea) was used to evaluated skeletal muscle mass including fat-free mass (FFM), soft lean mass (SLM), skeletal muscle mass (SMM), and skeletal muscle index (SMI).

### Cognition test

Cognition test was operated by a trained research staff member in a quiet test room. Global cognition was assessed using the Chinese (Mandarin) translation of the MoCA (Version 7) (from http://www.mocatest.org). The total score of the MoCA is 30 points and it covers seven components including visuospatial/executive functions (trail-making test: 1 point, copy tube: 1 point and clock drawing task:3 points), naming (3 points), attention (forward digit span: 1 point, backward digit span: 1 point, vigilance: 1point and serial 7 subtraction: 3 points), language (sentence repetition: 2 points, verbal fluency: 1 point), abstraction (2 points), delayed recall (5 points) and orientation (6 points). Higher scores in MoCA mean better cognitive performances.

### Plasma BDNF measurement

Human plasma BDNF concentrations were determined with an enzymelinked immunosorbent assay kit (Elabscience, E-EL-H0010) according to the manufacturer’s recommended instructions. The detection range was 31.25-2000pg/ml and the detection sensitivity of BDNF was 18.75 pg/ml. All plasma samples were measured at a dilution of 1:5.

### Plasma SIRT1 measurement

We detected plasma SIRT-1 using an enzyme-linked immunosorbent assay (ELISA) kit (Elabscience, E-EL-H1546). The detection range was 0.31–20 ng/ml and the sensitivity was 0.19 ng/ml. The intra and inter detection variability ranges are ≤ 8% and ≤ 12%, respectively. All plasma samples were measured at a dilution of 1:2.

### Follow-up and outcome

With a median follow-up of 1,136 days, participants were administered cognitive test. The primary outcome was cognitive decline using the change of MoCA. The cognitive decline defined as the difference of MoCA score at the 3 year and baseline over -2.

### Statistical analysis

Our data was analyzed using SPSS 25.0. Categorical variables were presented as frequencies and continuous variables are presented as mean ± standard deviation or median (interquartile range). Student t-test or Chi-squared test was used for the mean comparison between two independent groups. Univariate and multivariate Cox regressions were used to assess the relationship between cognition and muscle mass, with age, sex, body mass index, education year, diabetes, previous history of cardiovascular disease and stain included as covariates. Mediation analysis, which is a regression-based path analysis technique, was performed to examine the indirect effect of skeletal muscle on cognitive decline through BDNF. The statistical significance level was taken as 0.05.

## Results

### Baseline characteristics of the study population

​A total of 166 maintenance hemodialysis patients enrolled in the study between July 2017 and January 2018 were assessed at 3-year follow-up. The baseline characteristics of the patients are given in **Table 1**. The mean age was 49.9 ± 11.2 years, and there were 102 (61.4%) male participants. The average FFM was 44.8 ± 8.3 kg, SLM was 42.2 ± 7.9kg, SMM was 24.5 ± 5.0kg, and SMI was 6.7 ± 1.0kg/m^2^.

**Table 1:** Characteristics of the study population at baseline (N=166).

	Mean ± SD/Number (%)
Age, years	49.9±11.2
BMI, kg/m^2^	22.6±3.5
Male	102(61.4%)
Education year	10.0±3.4
Current smoker	46 (27.7%)
Diabetes	23 (13.9%)
Cardiovascular disease	6(3.6%)
Statins	4 (2.4%)
Systolic BP, mm Hg	138.4±17.7
Diastolic BP, mm Hg	84.5±11.8
Albumin, g/L	47.0±3.5
Hemoglobin, g/L	106.8±15.0
Total cholesterol, mmol/L	4.5±1.1
Triglycerides, mmol/L	2.1±1.3
HDL cholesterol, mmol/L	1.2±0.4
LDL cholesterol, mmol/L	2.4±1.0
Calcium, mmol/L	2.5±0.2
Phosphorus, mmol/L	1.7±0.4
SLM, kg	42.2±7.9
SMM, kg	24.5±5.0
FFM, kg	44.8±8.3
SMI, kg/m^2^	6.7±1.0

BMI, body mass index; eGFR, estimated glomerular filtration rate; HDL-C, high-density lipoprotein cholesterol; LDL-C, low-density lipoprotein cholesterol; SLM, soft lean mass; FFM, fat free mass; SMM, skeletal muscle mass; SMI, skeletal muscle index.

### Follow-up and endpoints

**Figure 1:**
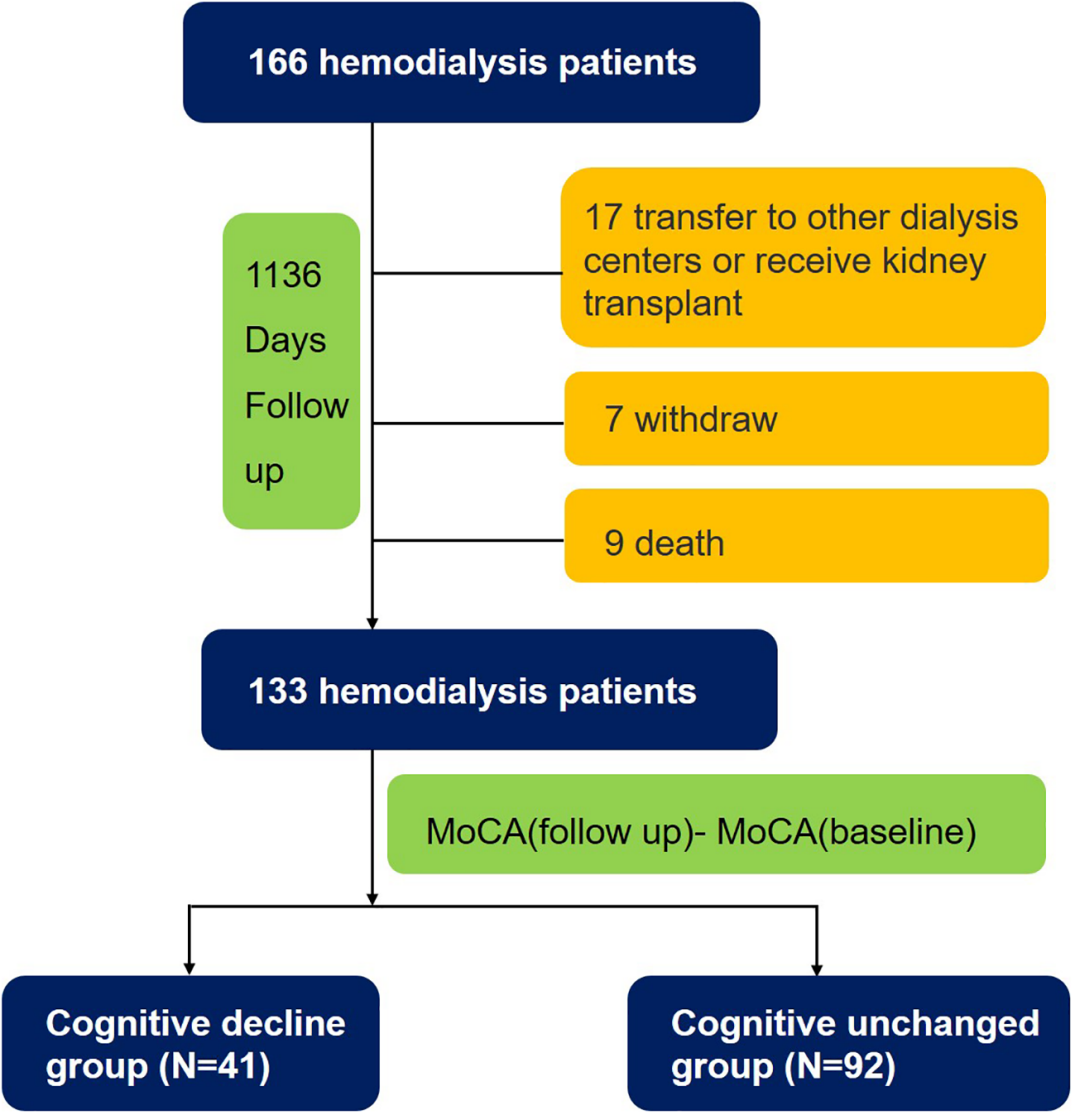
Flow-chart of the study.

**Figure 2:**
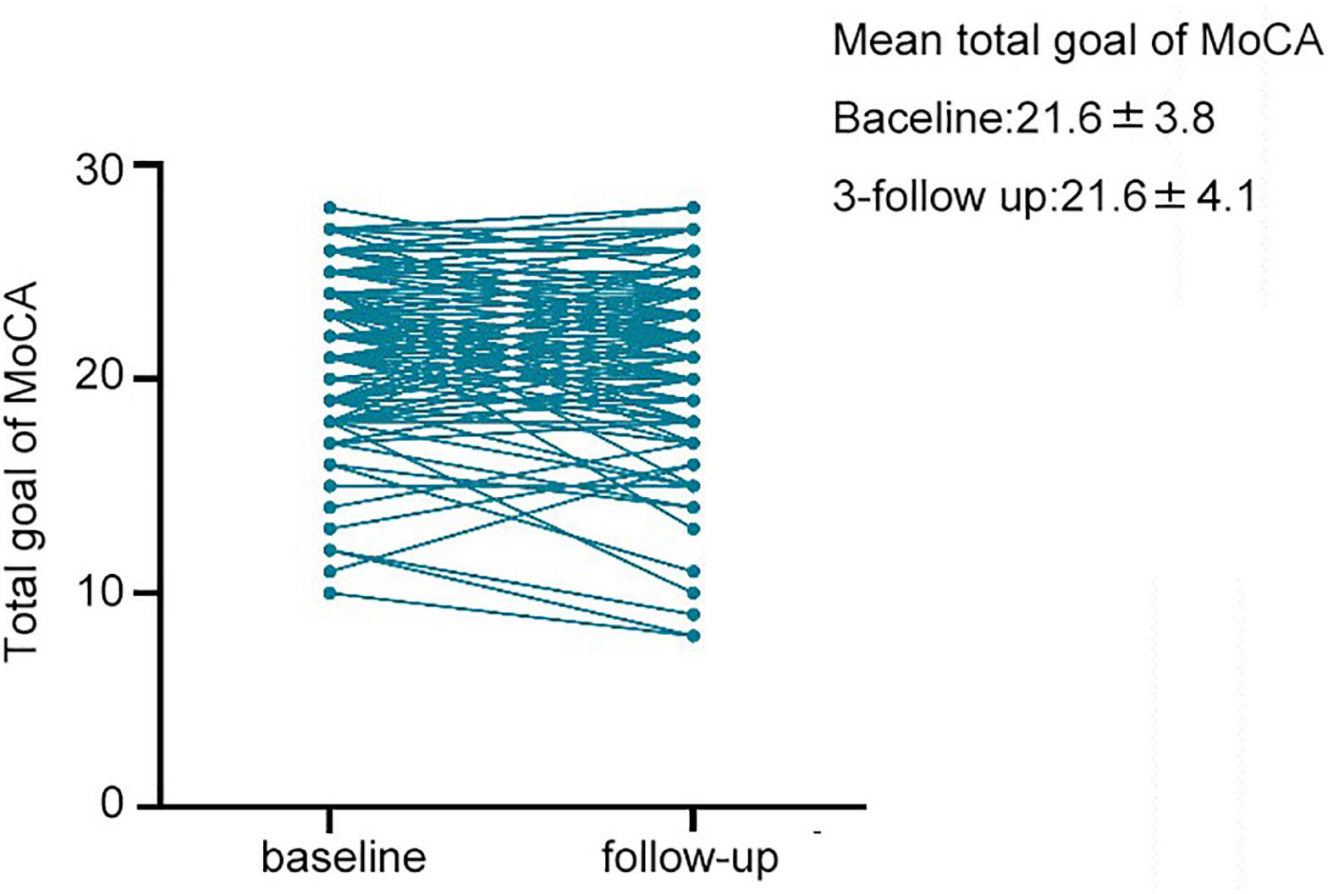
Total goal of MoCA at baseline and 3-year follow up. The mean MoCA score of patients in baseline and follow-up time was 21.6 ± 3.8 and 21.6 ± 4.3.

During a median follow-up period of 1,136 days, 9 subjects died, 24 subjects dropped out, and 133 (80.1%) subjects completed all tests. The patients were divided into two groups, the cognitively unchanged and cognitive decline groups, based on the difference in MoCA scores between the follow-up and baseline. Overall, 41 (31%) patients undergoing HD experienced cognitive decline (**Figure 1**). The mean MoCA scores of the patients at baseline and follow-up were 21.6 ± 3.8 and 21.6 ± 4.1 (**Figure 2**), respectively.

### Risk factor of cognitive decline

As shown in **Table 2**, compared to the cognitively unchanged group, the cognitive decline group were older (52.6 ± 10.3 vs 48.6 ± 11.4, P = 0.048) and had fewer years of education (9.1 ± 4.1 vs 10.5 ± 3.0, P=0.028). No significant differences were found in smoking, diabetes, BMI, albumin, or hemoglobin levels between the two groups. Patients in the cognitive decline group had lower SLM, SMM, FFM, and SMI (all P < 0.05) than those in the cognitively unchanged group. The adjusted HRs from the multivariate Cox models are presented in **Table 3**. SLM, SMM, SMI, and FFM were predictors of cognitive decline. Independent confounding variables included age, sex, years of education, body mass index, diabetes mellitus, previous history of cardiovascular disease, and stain (SLM, HR 0.932, P = 0.011; SMM, HR 0.903, P = 0.018; SMI, HR 0.63, P = 0.027; FFM, HR 0.935, P = 0.011).

**Table 2:** Risk factors of global cognitive decline.

	Cognitive unchanged (N=92)	Cognitive Decline (N=41)	*p*
Age, years	48.6±11.4	52.6 ± 10.3	0.048
Sex, Male	60(65.2%)	18(43.9%)	0.022
Education year	10.5±3.0	9.1 ± 4.1	0.028
Current smoker	25(27.2%)	8(19.5%)	0.347
Diabetes	10(10.9%)	5(12.2%)	0.824
Cardiovascular disease	4(4.4%)	1(2.4%)	0.594
Statins	3(3.3%)	1(2.4%)	0.798
BMI, kg/m^2^	22.7 ± 3.6	22.9 ± 4.0	0.789
Albumin, g/L	47.3 ± 3.7	46.4 ± 3.6	0.182
Hemoglobin, g/L	108.0 ± 13.6	105.2 ± 16.6	0.332
SLM, kg	42.8 ± 7.5	39.2 ± 7.4	0.011
SMM, kg	24.9 ± 4.8	22.6 ± 4.6	0.01
FFM, kg	45.4 ± 7.9	41.6 ± 7.8	0.011
SMI, kg/m^2^	6.8 ± 1.0	6.3 ± 1.0	0.018

Cognitive Decline defined as a follow-up score on MoCA that was 1/2 SD or more below the baseline.

**Table 3:** Associations between global cognitive decline and skeletal muscle indicators.

	HR	95% confidence interval	p
SLM, kg	0.932	0.882 – 0.984	0.011
SMM, kg	0.903	0.829 – 0.983	0.018
SMI kg/m^2^	0.63	0.418 – 0.950	0.027
FFM, kg	0.935	0.888 – 0.985	0.011

adjust for age, sex, education year, body mass index, diabetes mellitus, previous history of cardiovascular disease, stain.

### Mediating role of BDNF between skeletal muscle mass and cognitive impairment

**Figure 3:**
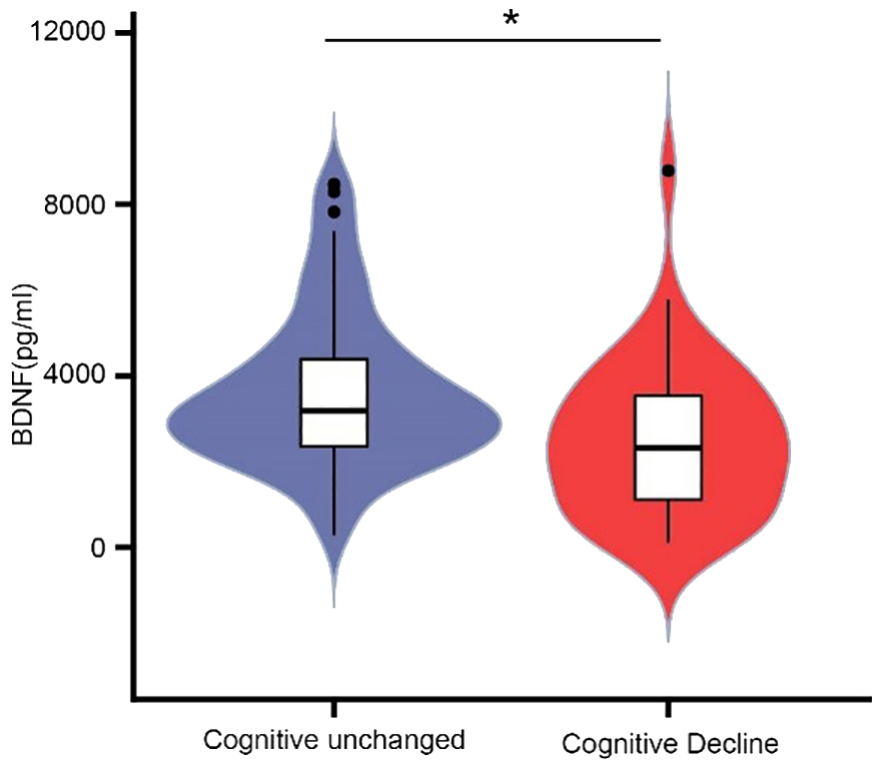
Comparison of plasma BDNF levels between cognitive unchanged and decline group. The mean BDNF level of the cognitive decline group was 2313.46(1039.42-3592.56) pg/mL, and 3182.87(2343.05-4459.37) for the unchanged group. * P<0.05.

To explore the association between muscle function and cognition, we measured plasma levels of BDNF. The mean BDNF level of the cognitive decline group was 2313.46 (1039.42-3592.56) pg/mL, while the level in the cognitively unchanged group was 3182.87 (2343.05-4459.37) pg/ml (P = 0.001) (**Figure 3**).

**Figure 4:**
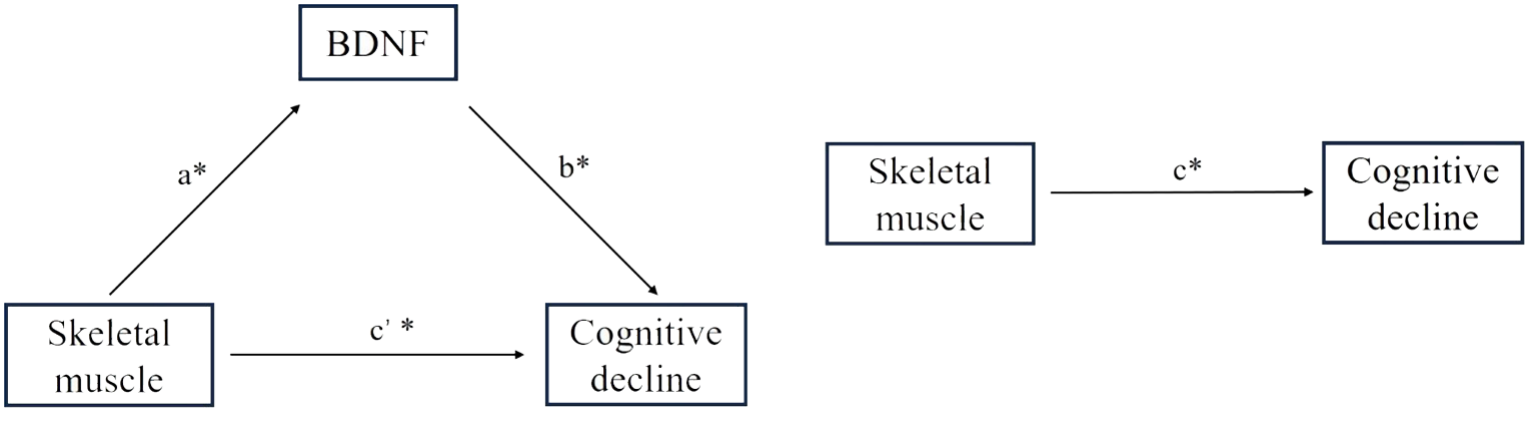
Effect of skeletal muscle on global cognitive decline through BDNF. a= Effect of skeletal muscle on BDNF; c’= Effect of indirect skeletal muscle on cognitive decline; c= Effect of direct skeletal muscle on cognitive decline; b= Effect of BDNF on cognitive decline. * P<0.05.

We hypothesized that BDNF levels may mediate the underlying mechanism of cognitive impairment in lower skeletal muscle mass. As shown in **Figure 4**, while the effect of skeletal muscle on BDNF was evaluated for the indirect or direct effect of skeletal muscle on cognitive decline was evaluated for c’ and c (all P < 0.05). The standardized regression coefficient (β) for the association between BDNF and cognitive decline (b) was 1 (P < 0.05). Therefore, BDNF had a partial direct effect on cognitive decline, and the contribution rates of the intermediary effect to the total effect were 21%, 19.7%, 35%, and 20.7% for SLM, SMM, SMI, and FFM, respectively (**Table 4**).

**Table 4:** The indirect effect of skeletal muscle on cognitive decline through BDNF.

	a	b	c	Effect M
SLM, kg	0.196	1	0.935	21.0%
SMM, kg	0.177	1	0.898	19.7%
SMI kg/m^2^	0.217	1	0.62	35.0%
FFM, kg	0.194	1	0.938	20.7%

SIRT1 has been reported to regulate BDNF expression and we further examined plasma SIRT1 levels (12). Mean plasma SIRT1 levels of the cognitive decline group were 1.85 (1.01-2.96) ng/mL, compared with 2.01 (1.38-3.47) ng/mL for the cognitively unchanged group, which was close to statistical significance (P = 0.059). SIRT1 levels were positively associated with BDNF levels (r = 0.273, P < 0.001).

## Discussion

This single-center prospective cohort study illustrated a correlation between decreased skeletal muscle mass and cognitive decline in patients undergoing hemodialysis. The results showed that 31% of the patients undergoing hemodialysis experienced cognitive decline during the 3-year follow-up period. Decreased muscle mass was found to be an independent risk factor of cognitive decline. In addition, patients with cognitive decline have significantly lower plasma BDNF concentrations than cognitively unchanged patients. Plasma BDNF levels account for 20%-35% of the association between skeletal muscle mass and cognitive function.

The kidneys and nervous system are interconnected through several pathways, and decreased renal function can lead to various pathological changes in the nervous system, including cognitive impairment (13). Cognitive impairment can occur in all stages of CKD. However, cognitive impairment mostly occurs in patients undergoing hemodialysis, affecting 70−90% of patients (1). The fluctuation in the prevalence of cognitive impairment is mainly due to the use of different cognitive assessment tools and criteria. Our study found that 31% of patients undergoing hemodialysis experienced a decline in total MoCA score of ≥2 points after the 3-year follow-up period compared with that at baseline, indicating cognitive decline. The criterion used in our study is inconsistent with other studies in that most used a cross-sectional MoCA score of < 26 as the criterion for cognitive impairment (14, 15). In contrast, our results were based on the patients’ baseline scores, and the change in individual scores at follow-up was used as the criterion to determine whether cognitive decline had occurred.

At present, the pathophysiological mechanisms underlying dementia and mild cognitive impairment (MCI) in patients with CKD are not fully understood; however, causes of cognitive impairment in CKD are certain to be multifaceted. The risk factors for dementia and MCI in the general population are common risk factors for cognitive impairment in people with CKD and include aging, low educational level, ethnicity, diabetes, hypertension, hyperlipidemia, anemia, obesity, chronic inflammation, and a number of genetic markers associated with cognitive impairment (16, 17). Our results confirmed that patients in the cognitive decline group were older, had a lower educational level, and consisted of a greater proportion of females than those in the group with normal cognitive function. However, no significant difference was observed for the proportion of patients with diabetes, possibly because both groups included only a small proportion of patients with diabetes.

Surprisingly, our results found a difference in skeletal muscle mass between the two groups and found that loss of muscle mass was an independent risk factor for the development of cognitive decline. In a cross-sectional study, Nourbashemi et al. examined the correlation between lean body mass and cognitive function in 7,105 women. The results showed that women in the lowest quartile of lean body mass had 1.43 times the risk of cognitive impairment than women in the highest quartile (18). Additionally, Kwak et al. demonstrated that older women with sarcopenia have extensive cranial nerve degeneration and a significantly higher risk of cognitive impairment than those in the control group (19, 20). Increasing skeletal muscle mass through exercise can significantly improve memory, processing speed, and executive function while decreasing the rate of cognitive decline in elderly people and children (21). However, Ishii et al. conducted a study involving an elderly population from a Japanese community, which yielded the opposite result, suggesting that lower extremity function, rather than skeletal muscle mass, was an independent risk factor for cognitive function (22). These conflicting results may be associated with the use of the Mini-Mental State Examination, which is less sensitive than the MoCA for assessing cognitive function. In addition, appendicular skeletal muscle mass was assessed by Ishii et al., whereas we evaluated the skeletal muscle of the whole body.

BDNF, a secreted growth factor belonging to the neurotrophin family, is widely involved in neurophysiological processes, including neurodevelopment, regulation of cytogenesis and synaptogenesis, and neuroprotection. It also influences memory and cognition. The human BDNF gene consists of 11 exons, and differential splicing allows the formation of different transcripts that are specific to different tissues and respond to different stimuli (23). Exercise can increase BDNF levels in the hippocampus of mice, thereby improving their performance in the Morris water maze test and learning-related behavioral tasks (24). Numerous clinical trials have also found that low plasma BDNF concentrations are associated with poor cognitive function in elderly women living in the community (25). Our findings are consistent with the aforementioned results, where we found that plasma BDNF concentrations were significantly lower in patients with cognitive decline than in those with unchanged cognitive function, suggesting that plasma BDNF levels are significantly associated with cognitive function in patients undergoing hemodialysis.

Muscle BDNF is primarily involved in autocrine and paracrine signal transduction, which promotes fat oxidation in muscle fibers and potential muscle development (26). Myokines secreted by muscle fibers not only act locally but also affect other organs and tissues, regulating peripheral metabolic and physiological functions through hormone-like signal transduction (27). Currently, multiple candidate molecules have been proposed to mediate brain-muscle communication. Skeletal muscles produce irisin, a myokine, by cleaving the fibronectin type III domain-containing protein 5 (FNDC5). Irisin binds to neuronal receptors and upregulates BDNF expression, thereby affecting neurogenesis and enhancing cognition (28). In contrast, the brain controls the relationship between skeletal muscle function and peripheral metabolism via direct innervation of target tissues and modulation of sympathetic tone through cortisol produced by the hypothalamic-pituitary-adrenal axis (29). The results of the mediation analysis further suggested that plasma BDNF accounts for 20–35% of the association between skeletal muscle mass and cognitive function. However, the signaling pathways through which BDNF mediates skeletal muscle and cognitive functions require further investigation using animal models.

Sirtuin-1 (SIRT1) is a histone deacetylase expressed in the brain, liver, skeletal muscle, and adipose tissue. It is involved in learning, memory, and emotional regulation by activating BDNF transcription and regulating BDNF expression (30). A previous study reported that the plasma concentration of SIRT1 was positively correlated with BDNF levels in patients with depression (31). Liu et al. found that SIRT1 mediates the BDNF–TrkB signaling pathway and participates in the mechanism of rat brain damage caused by fluorosis (32). We also observed that SIRT1 levels were positively correlated with BDNF levels. However, the effect of SIRT1 on BDNF requires further investigation.

This study has some limitations. First, we examined plasma BDNF concentrations at baseline but did not examine BDNF concentrations during the 3-year follow-up period. Second, we included study participants with a mean age of 49.9 years, who were younger than the general populations of many developed countries, and thus the results cannot necessarily be extrapolated to other hemodialysis centers. Finally, our sample size was relatively small. Hence, further multicenter cohort studies with larger sample sizes are required.

In conclusion, our findings demonstrate that plasma BDNF concentrations mediate the effect of skeletal muscle mass on cognitive function in patients undergoing hemodialysis. This study provides a partial understanding of the complex mechanisms driving muscle-brain communication and offers a potential preventive and therapeutic option for reducing cognitive decline in patients undergoing hemodialysis.

## Data availability statement

The original contributions presented in the study are included in the article/**Supplementary material**. Further inquiries can be directed to the corresponding authors.

## Ethics statement

The studies involving humans were approved by The Second Affiliated Hospital, Nanjing Medical University. The studies were conducted in accordance with the local legislation and institutional requirements. The participants provided their written informed consent to participate in this study.

## Author contributions

LW: Formal analysis, Writing – original draft, Software. XB: Data curation, Writing – review & editing. LL: Data curation, Writing – review & editing. QH: Methodology, Investigation. JX: Validation, Writing – review & editing. XC: Validation, Writing – review & editing. HY: Project administration, Writing – review & editing. JY: Funding acquisition, Writing – review & editing. LJ: Supervision, Writing – review & editing.

## Supplementary material

Supplementary Figure 1 Correlation between SIRT1 and BDNF. **(A)** The mean SIRT1 level of the cognitive decline group was 1.85(1.01-2.96) ng/mL, and 2.01 (1.38-3.47) for the unchanged group. P=0.059. **(B)** Pearson correlation between plasma SIRT1 level and BDNF. The SIRT1 level was logarithm transformed in Pearson’s correlation analysis due to skewed distribution.
