# Supplementary figures and images for: Association between cognitive function and skeletal muscle in patients undergoing maintenance hemodialysis
Source: Front Endocrinol (Lausanne). 2024 Mar 15;15:1324867. doi: 10.3389/fendo.2024.1324867 (PMC10981270; doi:10.3389/fendo.2024.1324867)

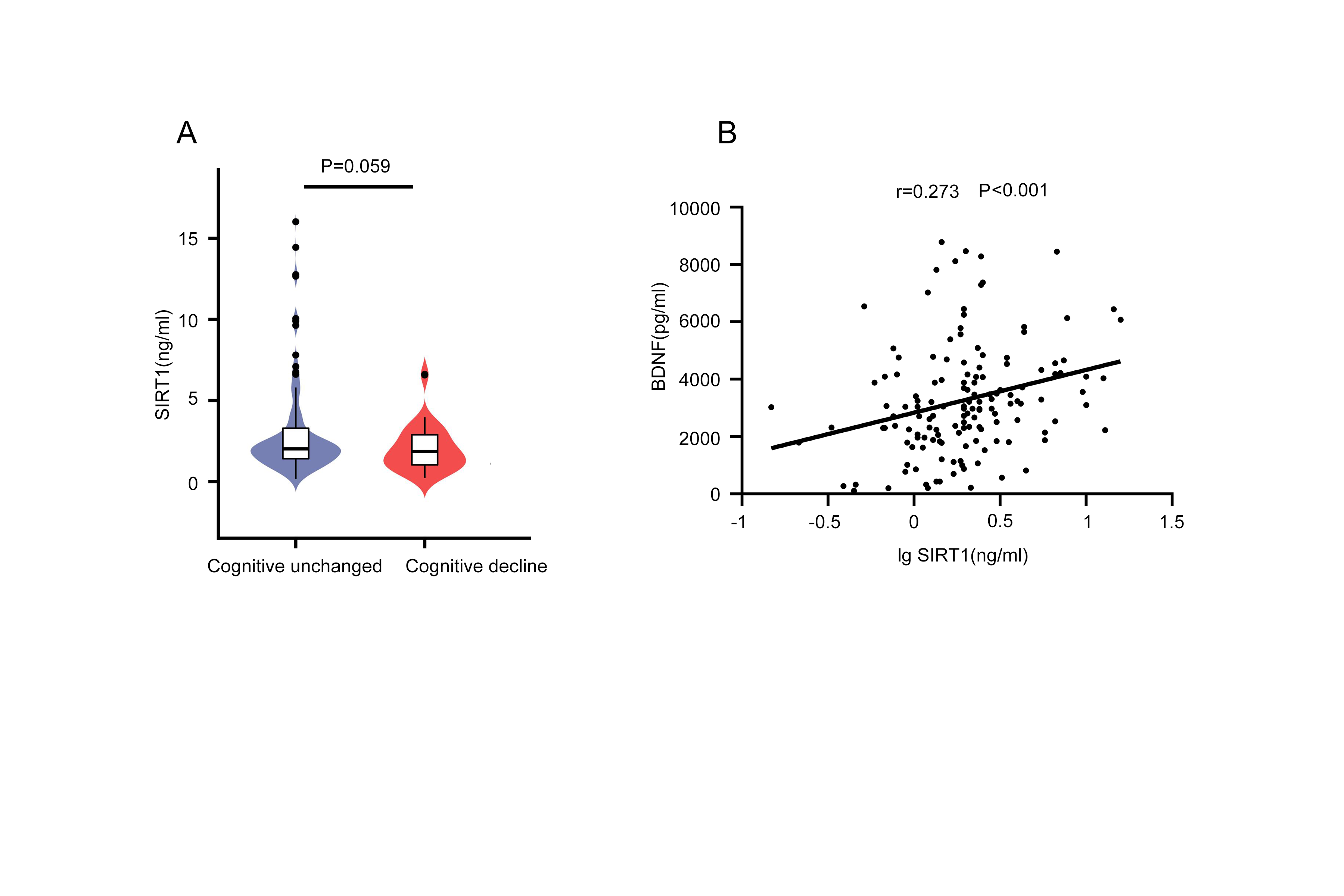

Supplement: Supplementary Figure 1 — Correlation between SIRT1 and BDNF. (A) The mean SIRT1 level of the cognitive decline group was 1.85(1.01-2.96) ng/mL, and 2.01 (1.38-3.47) for the unchanged group. P=0.059. (B) Pearson correlation between plasma SIRT1 level and BDNF. The SIRT1 level was logarithm transformed in Pearson’s correlation analysis due to skewed distribution. [file Image_1.tif]
